# *Psoralea karooensis* (Psoraleeae, Fabaceae): a new species from the Klein Karoo region of South Africa

**DOI:** 10.3897/phytokeys.17.3672

**Published:** 2012-09-12

**Authors:** Charles H. Stirton, A. Muthama Muasya, Jan Vlok

**Affiliations:** 1 Bolus Herbarium, Botany Department, University of Cape Town, Private Bag X3, Rondebosch 7700, South Africa; 2 Regalis, 102 Hope Street, Oudtshoorn 6620, South Africa

**Keywords:** Fabaceae, Klein Karoo, Leguminosae, New species, *Psoralea*, Psoraleeae, South Africa, Taxonomy

## Abstract

A new species of *Psoralea* is described. *Psoralea karooensis* C.H. Stirt., Muasya & Vlok is endemic to mountain streams in the Klein Karoo region of the Western Cape Province, South Africa. The new species is characterised by its flexuose habit of many stiff bare stems with the seasonal shoots arising apically in clusters and its greenish cream flowers borne at the apex of 10–12 mm long peduncles each ending in a trifid cupulum.

## Introduction

*Psoralea* L. comprises ± 70 species of mostly shrubs which are widespread in the winter rainfall area of South Africa and extend into Afromontane regions (Stirton 2005). The genusis commonly found in mountain fynbos in drainage systems (river beds, stream banks, seepage areas), occurring frequently on sandstone derived soils across the Cape Floristic Region (Stirton and Schutte 2000). However, there are a number of species, occurring marginally to the main generic distribution, that have adapted to surviving in drier conditions along the arid Fynbos-Succulent Karoo boundary (e.g. *Psoralea angustifolia* Jacq., *Psoralea glaucescens* Eckl. & Zeyh., *Psoralea tenuifolia* L., and *Psoralea verrucosa* Willd.). A new species is described here which occurs along seasonal freshwater streams in the Klein Swartberg and Anysberg Mountains (Vlok and Schutte-Vlok 2010).

## Species treatment

### Psoralea karooensis

C.H. Stirt., Muasya & Vlok sp. nov.

urn:lsid:ipni.org:names:77122079-1

http://species-id.net/wiki/Psoralea_karooensis

Plates 1 , 2

#### Latin.

Psoralea glaucescens *affinis, sed habitu cernuo caulibus nudis rigidis multis, brachyblastis vernalis ab apice fasciculatis; foliis 1-foliolatis; brachyblastis floriferis vernalis ramosis erectis brevibus; floribus viridi-cremeis parvis cupulo trifido parvo apice pedunculi 10–12 mm longi vexilloque macula nectarifera purpurea singula, venis purpurescentibus differt*.

#### Type.

**South Africa:** Western Cape, Ladismith (3320), Witteberg Private Nature Reserve, Driedamhoek (–AD), at end of jeep track, 33°20'11.7"S, 20°31'57.7"E, 20 February 2011, Muasya, Chimphango, & Stirton 5927 (holotype: BOL!; isotypes: BM!, GRA!, K!, MO!, NBG!, NH!, P!, PRE!).

#### Description.

*Habit*erect, willowy, flexuose branched shrub to 3 m tall, reseeder but coppices regularly once established. *Stems* 1(2), brownish-grey with scattered white lenticels, weakly fissured, bare of leaves except on the seasonal shoots which arise in a characteristic burst-branching; shoots coppice seasonally on old stems, leafy along their entire length, glaucous green with a whitish bloom, glabrous, with small raised crateriform glands. *Leaves* 1-foliolate, petiolate, stipulate, glandular, glabrous. *Leaf size* variable, being larger on water shoots from the rootstock (20–23 mm long, 2–3 mm wide), becoming smaller up the flowering shoots, dull glaucous green; apex rounded to acute; base cuneate; tip arching; petiole 1–2 mm long. *Stipules* 1–2 mm long, fused at base, rigid, triangular, semi-patent, 1-veined, rapidly senescent on flowering shoots. *Flowers* 10.0–12.5 mm long, greenish cream, borne in most axils of the upper half of the seasonal shoots, 1–3 per axil; pedicels 2 mm long. *Peduncles* 10–12 mm long, purple-tinged, terminated by a small tri-toothed cupulum; cupulum teeth equal, broadly triangular, acute, green, <1.0mm long. *Calyx* 5 mm long, pale green, sometimes flushed with purple, glabrous; teeth triangular, all 2 mm long, carinal tooth broadest, blunt, weakly ribbed, glandular; tube longer than the teeth, accrescent in fruit, persisting after fruits and seeds are shed. *Standard* broadly elliptic, 10 mm long, 10–12 mm wide; claw 2 mm long, almost tubular; cream, nectar flash purple rising from above the strongly developed auricles to the apex; inner face of the standard greenish, veins purple; apex emarginate but standard not fully reflexed. *Wing petals* 10–11 mm long, 4 mm wide; claw 2.5 mm long; locked into keel fold but not fused, slightly longer than the keel, petal sculpturing present, upper basal, comprised of 4–5 transcostal parallel lamellae. *Keel petals* 10 mm long, 3 mm wide; claw 5 mm long; apex deep purple. *Androecium* 9 mm long; tenth stamen free; sheath split adaxially, fenestrate. *Pistil* 9 mm long; ovary 1.5 mm long, sessile, glabrous, thickened before point of flexure, height of curvature 3 mm, erect, penicillate. *Fruits* ovate, indehiscent, wall scarious, 3.8–4.1 mm long and 2.2–2.5 mm wide at mid length. *Seed* 3.5–4.0 mm long and 2.1–2.2 mm wide at mid length, dark olive green, smooth, aril present and light olive green.

**Plate 1.**
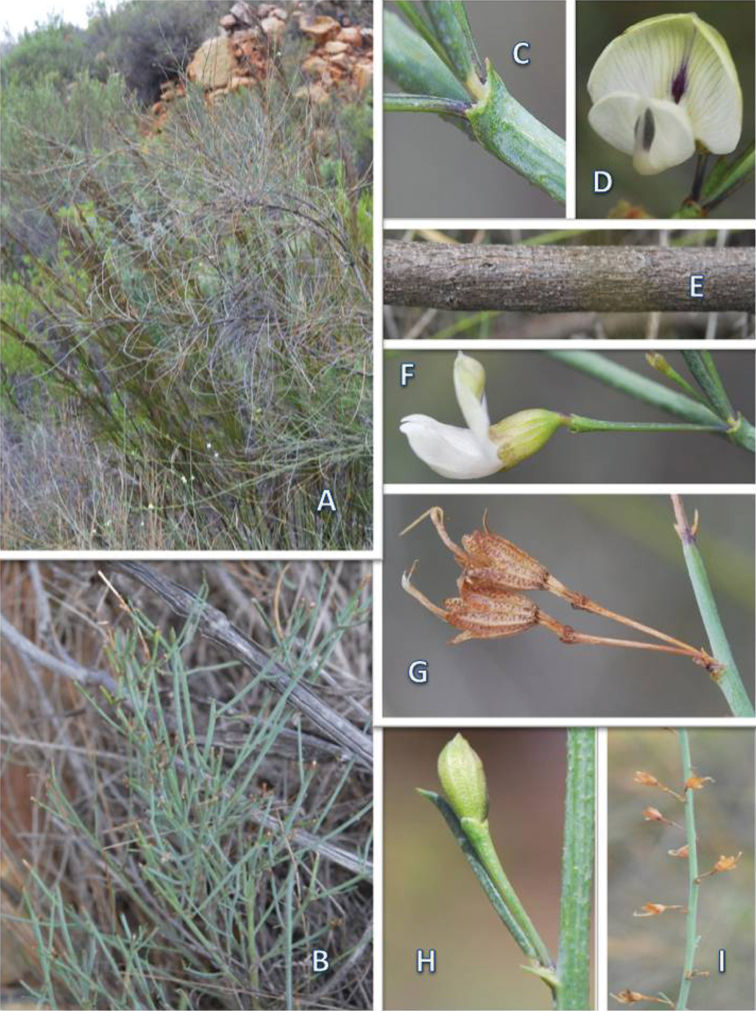
*Psoralea karooensis* C.H. Stirt., Muasya & Vlok **A** overseasonal shoots **B** seasonal shoots **C **fused stipules and base of leaflet **D** flower **E** bark **F** lateral view of flower, note small cupulum at base of calyx **G** persistent calyces after fruit and seed shed **H** flower bud and 1-foliolate leaf **I** fruiting branch. All photographs taken by CH Stirton. Voucher: Muasya, Chimphango, & Stirton 5927 (BOL).

#### Distribution and ecology.

**Plate 2.**
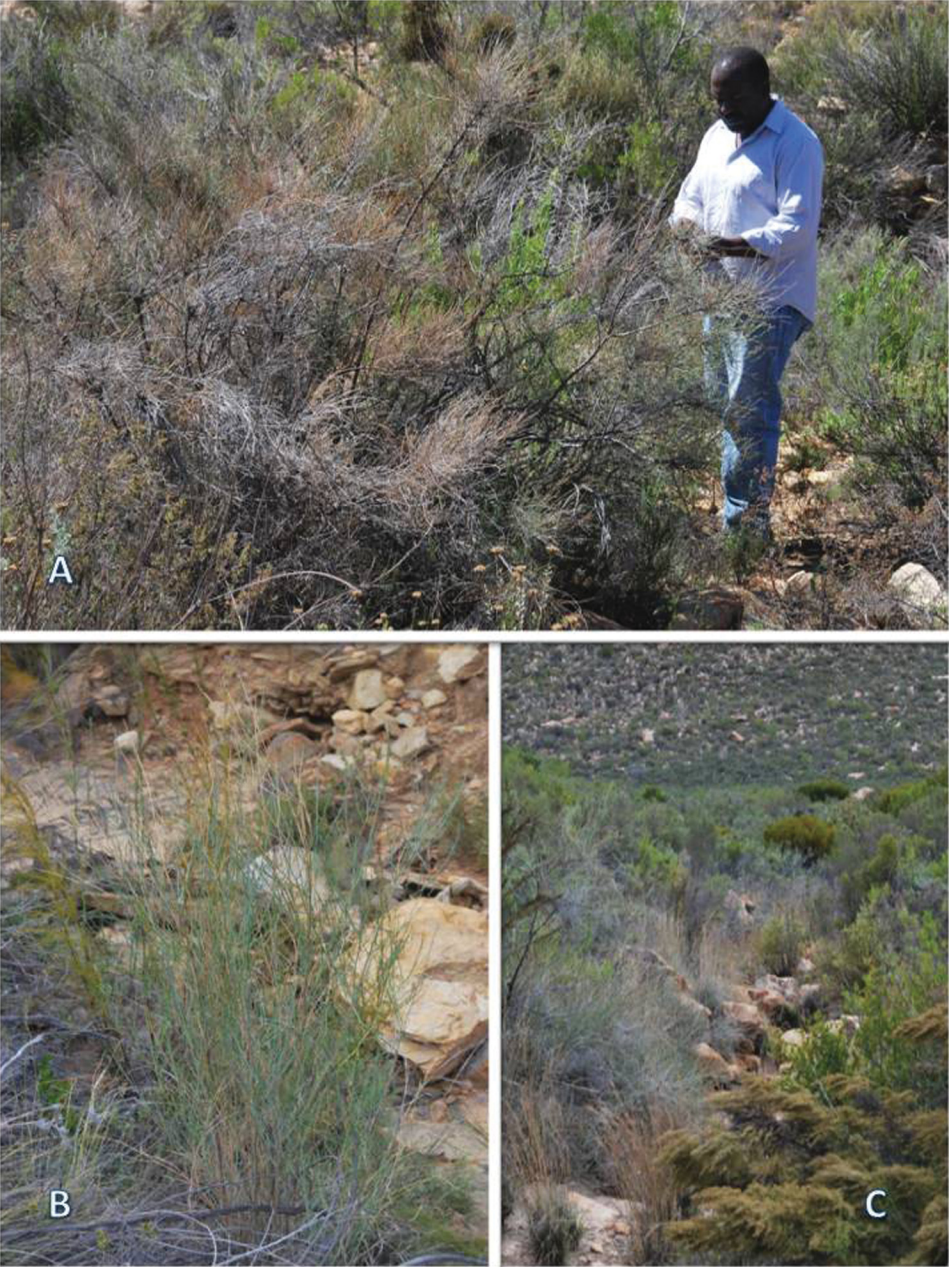
Habit and habitat of *Psoralea karooensis* C.H. Stirt., Muasya & Vlok **A** Mature plant **B** Young coppicing plant **C** typical dry gulley habitat. All photographs taken by CH Stirton. Vouchers: (**A**) Stirton, Muasya & Chimphango 13245 (BOL); (**B–C**) Muasya, Chimphango & Stirton 5927 (BOL).

*Psoralea karooensis* grows in the Klein Karoo mountains, between 1000–1200 m. It is restricted to the Witteberg quartzite fynbos vegetation, between the Witteberg and Anysberg Mountains, and occurs mainly in gulleys and along the banks of dry river beds (Plate 2).

#### Etymology.

The specific epithet alludes to its known restriction to the Klein Karoo.

#### Conservation status.

*Psoralea karooensis* is locally common within its range and much of its distribution occurs in protected nature reserves. However, the species is a habitat specialist (fresh water stream beds with intermittent flow) at above 1000 m and is only known from an area less than 500 km^2^. We therefore assess this species to be Rare under the South African Red list categories and criteria (EOO, AOO; Von Staden et al. 2009).

#### Other specimens examined.

**South Africa:** Western Cape: Laingsburg district (3320 AD), Elandsfontein Farm, on dirt road off N1 - Laingsburg via Witteberg [33°18'38.41"S, 20°26'46.13"E], 20 Feb 2011, *Stirton, Muasya & Chimphango 13245* (BOL); Witteberg Kloof, 16 Jul 1923, *Compton 2592* (BOL). Witteberg, S. base of the range, 31 Jan 1961, *Esterhuysen 28844* (BOL, PRE).

Western Cape: Ladismith district (3320 AD), 9 Aug 1939, *Levyns 6106* (BOL).

Western Cape: Laingsburg district, at entrance to Perdekloof Gorge, S. of Matjiesfontein at northern base of Witteberg (3320 BC), *Helme 2938* (NBG).

#### Discussion.

*Psoralea karooensis* is characterised by its small (< 13 mm long) greenish cream flowers with a small (< 1.0 mm long) trifid cupulum at the apex of a 10–12 mm long peduncle, standard with single purple flash nectar patch and purplish veins, 1-foliolate leaves, erect multi-branching short seasonal flowering shoots, and flexuose habit of many stiff bare stems with the seasonal shoots arising apically in clusters. It is most similar to *Psoralea glaucescens* Eckl. & Zeyh., a widespread but rare species found in the northwestern Cape. The resprouter *Psoralea glaucescens* differs in its densely branched mounded habit, grey puckered stems, 3–5-foliolate irregularly sized leaves, greenish yellow flowers, with violet veining and nectar flash, borne 1–5 per axil, and purplish calyces.

## Supplementary Material

XML Treatment for Psoralea karooensis

## References

[B1] Stirton CH (2005) Tribe Psoraleeae. In: Lewis G Schrire B Mackinder B Lock M (Eds). Legumes of the World.Royal Botanic Gardens, Kew: 447-451

[B2] Stirton CH Schutte AL (2000) *Psoralea*. In: Goldblatt P Manning JC (Eds). , Cape Plants: a Conspectus of the Cape Flora of South Africa.National Botanical Institute of South Africa, Pretoria: 505-507

[B3] Vlok J Schutte-Vlok AL (2010) Plants of the Klein Karoo. Umdaus Press, Hatfield, 412–415.

[B4] Von Staden L Raimondo D Foden W (2009) Approach to Red List Assessments. In: Raimondo D Von Staden L Foden W Victor JE Helme NA Turner RC Kamundi DA Manyama PA (Eds). Red List of South African Plants.South African National Biodiversity Institute, Pretoria: 6-18

